# Intra-MRI extraction of diagnostic electrocardiograms using dynamic feedback from carotidal magnetohydrodynamic voltages

**DOI:** 10.1186/1532-429X-18-S1-P214

**Published:** 2016-01-27

**Authors:** Thomas S Gregory, Kevin J Wu, Ehud J Schmidt, John Oshinski, Zion T Tse

**Affiliations:** 1 grid.213876.9000000041936738XCollege of Engineering, University of Georgia, Athens, GA USA; 2 grid.62560.370000000403788294Radiology, Brigham and Women's Hospital, Boston, MA USA; 3 grid.412162.20000000404415844Radiology, Emory University Hospital, Atlanta, GA USA

## Background

During Cardiac Magnetic Resonance Imaging (CMR), blood plasma electrolytes ejected into the aorta during early systole interact with the static magnetic field of the MR scanner (B_0_) to produce a Magnetohydrodynamic (MHD) Effect [1]. Electrocardiograms (ECGs) recorded in the presence of B_0_ are overlaid with induced MHD voltages (V_MHD_), leading to non-robustly synchronized imaging [2], and preventing reliable physiological monitoring inside the MRI [3]. Previous methods have sought to separate between V_MHD_ and the true ECG (ECG_real_) through adaptive filtering [3], independent component analysis [4], and advanced computational models [5]. However, these methods are based on a static model, which has limited accuracy during varying-rate heart-beats. We aim to develop accurate ECG_real_ extraction, as well as real-time Stroke Volume (SV) estimation (proportional to the integral of MHD over systole) [6], with the advantage of physiological feedback through the real-time monitoring of common carotidal MHD, through which the previously static MHD template can be dynamically updated, providing an increased level of accuracy during variations in heart rate, and a continuous estimation of V_MHD_ and ECG_real_, for the patient's entire duration inside the MRI.

## Methods

**Figure 1:**
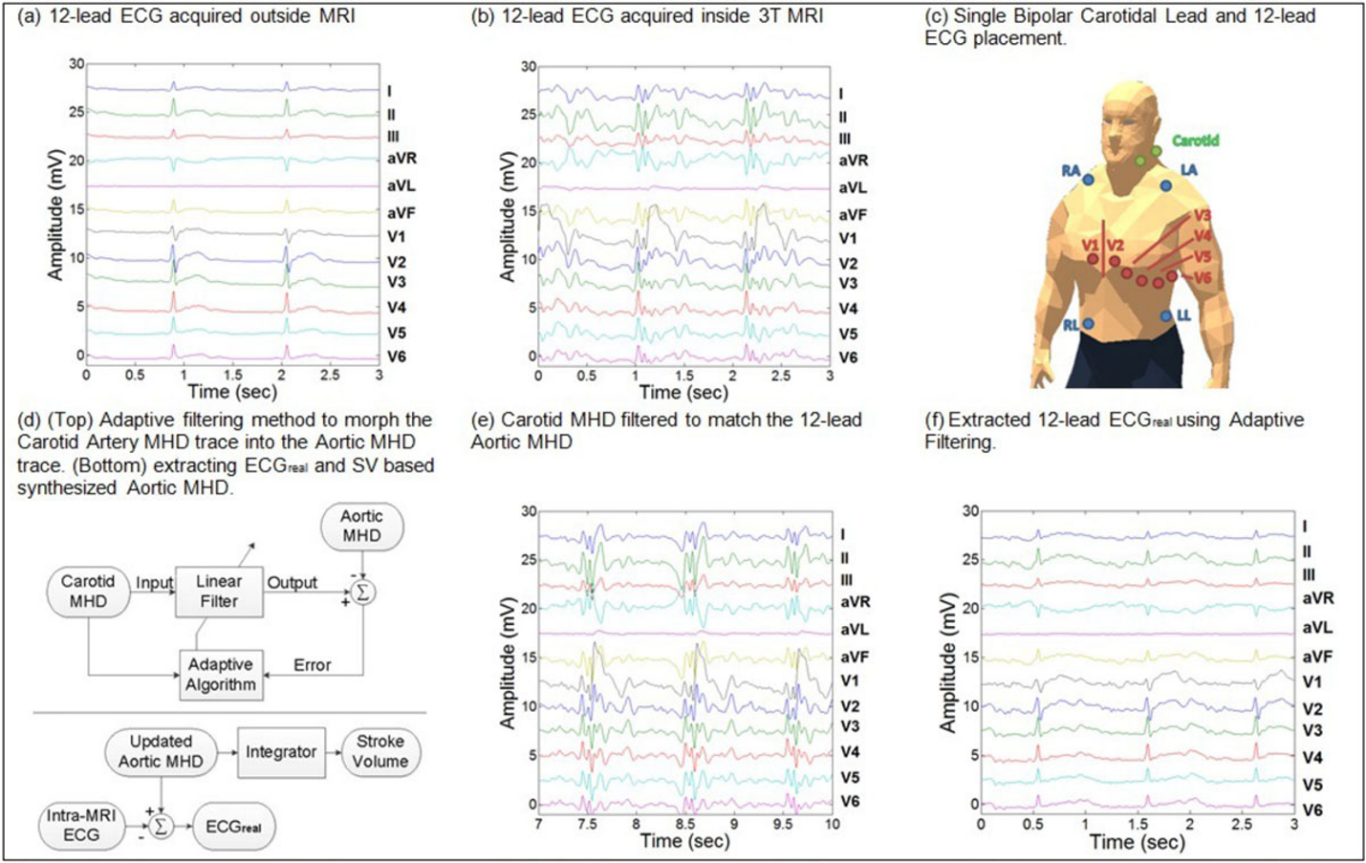
**Active removal of induced Magnetohydrodynamic voltages in ECGs recorded inside a 3T MRI using adaptive filtering**.

12-lead ECGs were acquired in two (n = 2) healthy volunteers during 20-second breath-holds in a 3T MRI (Figure 1ab) with the heart at isocenter. A secondary monitor was used to acquire a single anterior-posterior bipolar lead placed approximately on the left common carotid artery (Figure 1c). ECGs were acquired inside (ECG_real_ + V_MHD_) and outside (ECG_real_) the MRI bore during an initial phase in which a static MHD template was extracted, based on lead subtraction. Carotidal MHD was extracted from the single bipolar lead and phase-compensated to match V_MHD_ obtained from the 12-lead ECG. Carotid MHD was subsequently used to adaptively train a Least Mean Squares filter (Figure 1d) to update the MHD template and produce: (1) clean 12-lead ECGs; and (2) an accurate SV estimate [6] (Figure 1e).

## Results

The adaptive filtering method was shown to reduce V_MHD_ in the acquired 12-lead ECGs, with residual noise forming <5% of the R-wave amplitude. The method preserved the true S-T segment, while requiring only a short training phase for the 12-lead ECG (10-15 seconds). The Pearson's Correlation Coefficient between Aortic and Carotid MHD increased from 0.51 to 0.88 after the adaptive filtering routine was applied. Figure 1f shows the extracted 12-lead ECG acquired inside the MRI bore after the training phase.

## Conclusions

A method to extract true sinus rhythm beats from intra-MRI 12-lead ECGs was presented and shown to provide accurate dynamic measurements of induced V_MHD_ using Carotid artery MHD and ECG_real_ to allow for advanced physiological monitoring inside the MRI.
